# Seed quality and seed quantity in red maple depends on weather and individual tree characteristics

**DOI:** 10.1002/ece3.6900

**Published:** 2020-10-14

**Authors:** Abigail R. Goszka, Rebecca S. Snell

**Affiliations:** ^1^ Department of Environmental and Plant Biology Ohio University Athens OH USA

**Keywords:** elevation gradient, embryo development, endosperm, seed fill, seed production

## Abstract

Under future climate change, plant species are expected to shift their ranges in response to increasing temperatures and altered precipitation patterns. As seeds represent the single opportunity for plants to move, it is critical to quantify the factors that influence reproduction. While total seed production is clearly important, seed quality is equally as critical and often overlooked. Thus, to quantify how environmental and tree‐level characteristics affect seed quality and quantity, the reproductive output of red maple (*Acer rubrum*) was measured along an elevation gradient in the Monongahela National Forest, WV. A variety of individual‐level characteristics were measured (e.g., DBH, canopy area, height, and tree cores were taken to quantify growth), and seed traps were placed under seed‐bearing trees to collect samaras and quantify total seed production. A random subsample of collected seeds from each tree was micro‐CT scanned to determine embryo volume, photographed for morphology measurements, and used for germination trials. The number of seeds produced was negatively affected by frost events during flowering, and stand density. The trees with the most seeds also showed reduced growth in recent years. Only 63% of scanned seeds showed embryo development, and of those seeds—only 23% germinated. The likelihood of embryo presence increased as growth rate decreased, while embryo size increased with tree height, smaller DBH, and in areas dominated by hemlock. Both larger embryo volume and larger overall seed size increased the likelihood of germination. The results highlight the importance of including seed quality in addition to seed quantity for a more complete representation of reproductive output.

## Graphical abstract

Seed production and germination are critical processes for the ability of plants to respond to climate change. We measured both seed production and seed quality for red maple along an elevation gradient. We established that seed production alone is not an accurate measure for reproduction, as only 63% of seeds contained embryos and embryo volume was the only factor which influenced germination success. Individual tree characteristics (e.g., DBH, age, height, and growth rate) were more important for predicting seed quality and quantity than weather. In fact, the only weather variable which was significant was the number of frost‐free days during flowering, which had a positive impact on number of seeds produced.

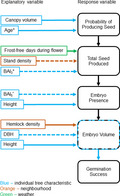

## 1 INTRODUCTION

In the northeastern United States, the last few decades have seen increases in mean annual temperatures and annual precipitation sums (Grundstein, 2008). In response to these ongoing and future climate changes, species are expected to shift their ranges to remain within their bioclimatic niche. Although northward range shifts have already been recorded for many short‐lived, highly mobile species, such as bird and butterfly species (Parmesan et al., 1999; Thomas & Lennon, 1999), not all species will be able to track rapid climate change. Sessile species with long generation times, such as trees, are likely to experience difficulty migrating quickly enough under future climate change. For example, empirical data from the Forest Inventory and Analysis program found that the majority of tree species in the eastern United States are experiencing range contractions at both their northern and southern range limits (Zhu et al., 2012). A comparable study in the western United States found a similar result, where 14 out of 18 trees showed range contractions instead of expansions (Bell et al., 2014). These studies suggest that we possess an incomplete understanding of the factors the influence plant migration.

Range shifts assume that trees can produce a large number of quality seeds that are successfully dispersed into new habitats, and will subsequently establish (Bykova et al., 2012; Clark et al., 1998). Thus, it is crucial to understand what factors influence both seed production and seed quality. Seed production has a strong positive correlation to basal area at the stand level (Clark et al., 1998; Kroiss & HilleRisLambers, 2015; Sakai, 1990), and at the individual level older, and larger individuals tend to produce more seed (Viglas et al., 2013). Extreme weather events, such as frost days during flowering or heavy winds during flowering and fruiting, can also have a negative impact on seed production (e.g., Tremblay et al., 2002). These results would suggest seed production might be limited at the edges of a species range, due to low parent tree abundance, younger or smaller trees, and/or more extreme weather.

Several studies of trees at the edges of their ranges have demonstrated reduced seed set, lack of seed maturation, lower seed mass, and reduced viability(Barclay & Crawford, 1984; Cuevas, 2000; Oleksyn et al., 1998; Pigott & Huntley, 1981; Rasmussen & Kollmann, 2004; Sirois, 2000; Sveinbjornsson et al., 1996). For example, *Picea mariana* and *Nothofagus pumilio* at the edge of their range showed a decrease in seed fill and germination (Cuevas, 2000; Sirois, 2000). However, other studies have found either no change in reproductive capacity or even increased seed set, seed maturation, seed mass, and germination/viability (Ettinger & HilleRisLambers, 2017; Kollas et al., 2012; Marcora et al., 2008). For example, a European study of species in the *Acer*, *Fagus*, *Fraxinus*, *Ilex*, *Laburnum*, and *Quercus* genera showed no change in seed quality over an elevation gradient (Kollas et al., 2012). *Betula* species increased in seed mass at higher elevations, but also experienced lower germination rates (Holm, 1994). Conditions at the edges of species ranges can influence plant reproduction; however, species‐specific relationships between weather and seed production remain unclear.

Predicting how climate change may impact seed production, and ultimately plant migration, requires a more holistic view. Plant reproduction is affected by weather at multiple stages (i.e., flower development, gametophyte development, pollination, and seed maturation; Bykova et al., 2012); thus, there are multiple opportunities for weather to impact both the total amount of seed and seed quality (i.e., embryo development and size). Empty seeds are commonly found in wild populations and may indicate a lack of available resources during seed filling (Fuentes & Schupp, 1998). In addition to the genetic component, environmental conditions during seed development can also lead to differences in seed quality by directly limiting the accumulation of dry matter, or indirectly by limiting the ability of the plant to produce the raw materials needed. For example, studies of *Parthenium hysterphorus* and *Anemone nemorosa* found that seed production and seed fill of an individual plant increased when grown under warm, wet conditions (De Frenne et al., 2010; Nguyen et al., 2017). However, in soybeans, increased temperatures during seed filling resulted in reduced seed quality, leading to lower germination and seedling growth (Spears et al., 1997). Although embryo development studies for tree species are relatively rate, similar patterns about the relationship between seed development and the environment have been found. *Sorbus torminalis* had a smaller seed set and a lack of seed maturation (i.e., low seed quality) at the edges of its range (Rasmussen & Kollmann, 2004), which was hypothesized to be from inbreeding depression, a lack of pollen, and/or low temperatures during fruit production. Another study that examined embryo development in *Tilia cordata* found that populations at the northern edge of their range, produced fruits that did not have fully developed seeds due to fertilization failure at temperatures below 15°C (Pigott & Huntley, 1981). Thus, environmental conditions can determine whether a seed has an embryo, as well as the size of the embryo, which can affect germination success and seedling vigor. Quantifying embryo size may provide a more accurate estimate for the percentage of the seeds produced by an individual that have the potential to germinate. By ignoring embryo presence and size, seed production estimates may be overestimating the number of viable seeds available for migration (Martyn et al., 2009).

Factors that influence seed viability need to be examined in addition to total seed production to better predict demographic changes and range shifts (Bykova et al., 2012; Keith et al., 2008). Thus, to accurately estimate the amount of seed available for migration events at the edge of their ranges, three questions must be addressed: (a) are the plants at the edges of their ranges producing seed? (b) how many seeds are produced? and (c) how many of those seeds have the potential to germinate? We chose to use red maple (*Acer rubrum* L.) as a study species to address these questions. Red maple is one of the most abundant species in eastern United States forests (Abrams, 1998) and is expected to shift its range in response to future climate change. More specifically, red maple growth and recruitment is projected to increase in the northern edges of its range, while growth will be reduced at its southern limits due to limited summer water availability (Zhang et al., 2015). Red maple has also been shown to have morphological changes in response to climate, as red maples in colder climates had larger leaves with more teeth, and were more dissected (Royer et al., 2008). Thus, we might expect reproductive characteristics to also be sensitive to climate. Finally, we chose red maple because it is not a hard mast species. Red maple consistently produces seeds every year, allowing us to address our research questions within a single year, instead of across multiple years. Specifically, this manuscript addressing the following research questions using red maple as a study species:

1. What factors determine the likelihood of a tree producing seed?
2. What factors influence the total number of seed an individual tree produces?
3. What factors impact the quality of a seed, and therefore its probability of successful germination?

## 2 MATERIALS AND METHODS

### 2.1 Study area and species

We measured red maple from two sites in the Appalachian Mountains, West Virginia, to capture a range of elevation. The lower site was located in the Fernow Experimental Forest (39°4'34.14"N, 79°39'12.76"W) and had a minimum elevation of 470 m a.s.l and a maximum elevation of 678 m a. s. l. The higher site was located approximately 6 km south in the Monongahela National Forest (39°1'11.81"N, 79°41'15.86"W) and ranged in elevation from 824 to 1,009 m a. s. l. The mean annual temperature in this area is 9°C with an average precipitation of 145 cm. The average temperature difference between the lowest and highest elevation (i.e., from 470 to 1,009 m) is ~3°C. The soil at both sites is loamy‐skeletal and a mixture of Typic Udorthents and Typic Dystrudepts. Both sites are secondary‐regrowth mixed hardwood forests and included other tree species such as *Carya ovata* (Mill.) K. Koch, *C. tomentosa* (Lam.) Nutt., *C. glabra* (Mill.) Sweet, *Acer saccharum* Marshall, *Tilia Americana* L., *Magnolia* spp., *Tsuga canadensis* (L.) Carriere, *Quercus rubra* L., *Q. alba* L., *Q. prinus* Nutt., and *Q. velutina* Lam.

Red maple is a generalist species with a range that covers most of eastern North American with the northern limit at about 48° latitude (Tremblay et al., 2002). Red maple has a broad elevation range and can be found growing from sea level to approximately 900 m a.s.l. (Burns & Honkala, 1990). Although it grows across a wide range of climatic conditions, soil types, and moisture levels, it does best on moderately well drained, mesic sites (Burns & Honkala, 1990). The red flowers appear before bud break in April to May, making it one of the first tree species to flower. The species is polygamodioecious with a labile sex expression (i.e., individual trees may be functionally male, female, or bisexual and some of them can change sex in different years). The seeds are double samaras that are dispersed primarily by wind after ripening in May to June, and may be secondarily dispersed by rodents (Sakai, 1990). Trees become reproductively mature as young as four years old, and trees 5–20 cm diameter at breast height (DBH) have been shown to produce upwards of 12,000 seeds per tree (Burns & Honkala, 1990).

### 2.2 Field methods

Potential study trees were identified in March and April of 2018 and were classified as seed‐bearing or non‐seed‐bearing in May. A total of 44 seed‐bearing trees were identified. To compare traits that influence the likelihood of a tree to produce seed, we randomly selected 44 out of the 136 nonseed‐bearing trees. There were 15 seed‐bearing and 15 nonseed‐bearing trees located at the low site and 29 seed‐bearing and 29 nonseed‐bearing trees at the high site. Seed‐bearing trees were given a visual score of seed development before dispersal. The scoring system was as follows: less than 20% of the canopy having seed = 1, 20%–80% of the canopy having seed = 2, and greater than 80% of the canopy having seed = 3. These broad categories were intended to reflect “low,” “medium,” or “high” producers, and ended up correlating well with the number of seeds collected. Under the canopy of each seed‐bearing tree, two 0.25 m^2^ seed traps were suspended one meter above the ground. The seed traps were checked weekly from May 18 to July 3, 2018.

For each of the 88 study trees (i.e., seed‐bearing and nonseed‐bearing), we measured DBH, canopy volume, tree height, basal area increment, age, and stand density. Canopy volume was estimated from crown height (measured with a Haglöf Vertex Laser Geo Hypsometer) and canopy spread (measured using the spoke method) using the calculation put forth in Thorne et al. (2002):

$$\text{Canopy}\;\text{volume}={\left(\text{crown}\;\text{diameter}\right)}^{2}\times \text{crown}\;\text{height}\times \text{shape}\;\text{parameter},$$

where the shape parameter was set to 0.5891 for an elongated spheroid crown shape. Tree height was measured using the hypsometer. Stand density (as a proxy for competition) was measured using a wedge prism with a count figure of 10. Using a wedge prism, four basal area measurements were taken (one for each cardinal direction) at the base of the study tree, these were averaged to get the basal area per hectare of the surrounding stand. Using a 4.3 mm (0.169 inch) increment borer, two tree cores were taken from each tree at breast height, for age and growth measurements.

### 2.3 Laboratory methods—seeds

**FIGURE 1:**
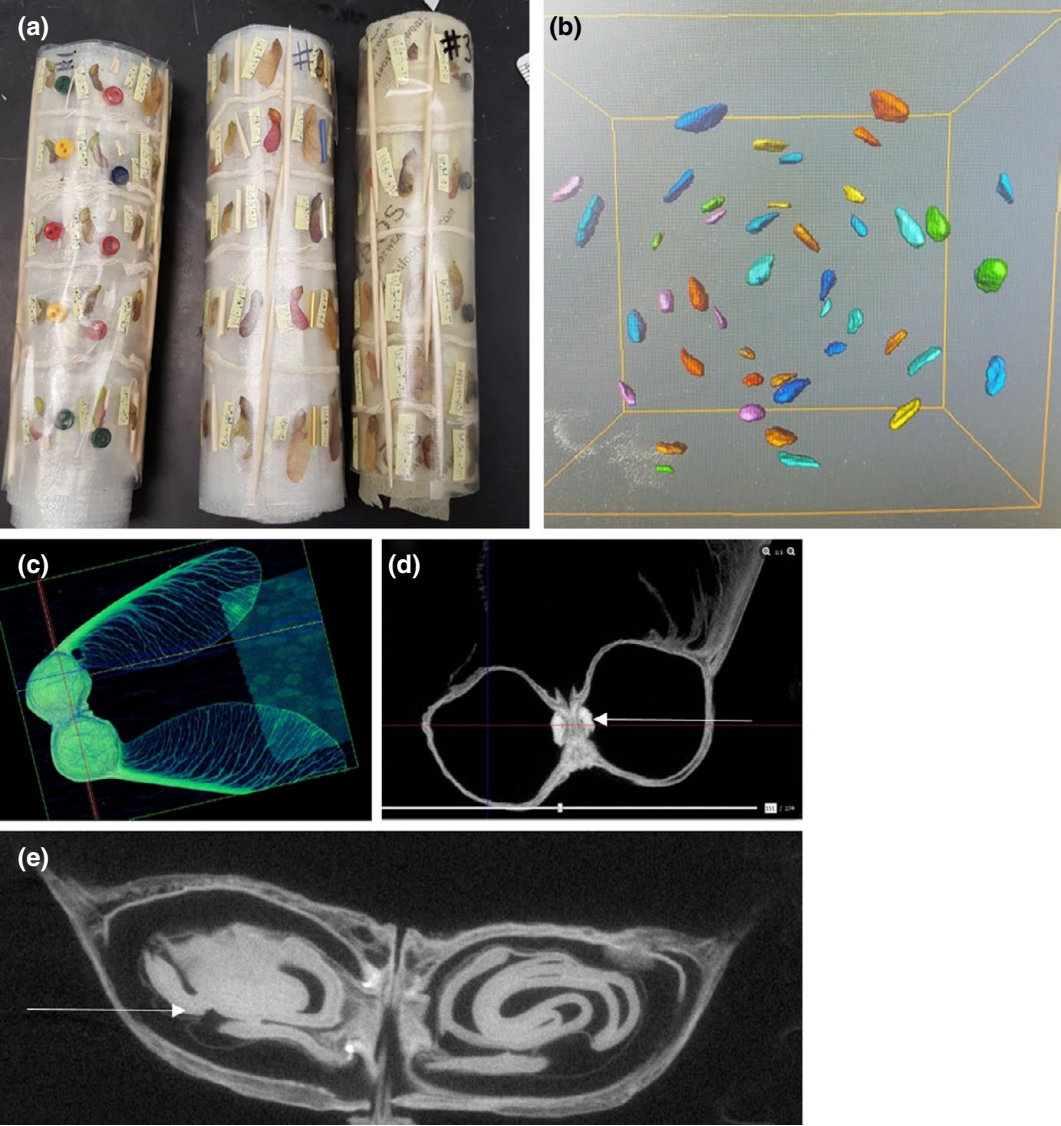
Images illustrating the micro‐CT scanning of seeds to measure embryo presence and embryo volume. (a) Three scan packets, each packet held approximately 220 red maple seeds. (b) Part of one of the scans, showing the red maple embryos. Panels (c), (d), and (e) are for silver maple. The white arrows are pointing to the embryo inside the seed cavity. Seed scan images are from the imaging software Avizo/Ameria v9.5

After collection, seeds were sorted into three categories: aborted, depredated, and fully formed. Aborted seeds were not fully formed (i.e., no wing development) and collected early in the season, depredated seeds had been chewed open, and fully formed seeds were intact seeds collected at the time of natural dispersal. From each seed‐bearing tree, 25 fully formed seeds were randomly selected for additional measurements. If the number of fully formed seeds collected from an individual tree was <25, then all of the fully formed seeds were used. Of the 44 seed‐bearing trees, we were unable to collect seeds from nine trees. These nine trees had all been scored as a 1. This gave a total of 662 study seeds from 35 trees. The selected seeds were weighed individually and placed in a scan packet and CT scanned using the TriFoil Imaging eXplore CT 120 Small Animal X‐Ray CT Scanner in the Edison Biotechnology Institute at Ohio University. The 3‐dimensional images produced from the CT scan were used to measure the volume of the embryo and seed cavity (mm^3^) with the imaging software Avizo/Ameria v9.5 (Figure 1).

After scanning, the seeds were digitally photographed and the length of the seed, width of the seed, area of the samara wing, and length of the entire samara (i.e., from the top of the seed to the bottom of the wing) were measured in ImageJ v1.5a. After being photographed, the seeds were placed in petri dishes on a thin layer of vermiculate and wetted with distilled water. The seeds were cold stratified in the fridge for a month and then placed in germination chamber on a diurnal setting (12 hr of light at 25°C and 12 hr dark at 13°C). The dishes were checked and rotated daily from January 14 to March 1, 2019. Germination was considered successful at the emergence of a radical root and then removed from the chamber.

### 2.4 Laboratory methods—tree cores

Tree cores were mounted on 2.54 × 2.54 cm core mounts using Elmer's glue and sanded using the guidelines presented by Spear (2010). The cores were aged by dot dating and skeleton plotting. Ring width for the last 5 years was measured using an Acu‐Rite Velmex and JX2 v4.1.2. The ring width data were then used to calculate the sum of the basal area increment for the past 3 years (BAI3) and 5 years (BAI5).

### 2.5 Data analysis

Twenty‐five temperature loggers were placed near study trees and recorded temperatures every 2 hr from March to October 2018. We used elevation to extrapolate temperature data from the loggers to the individual study tree. For each tree, we calculated the average, maximum, and minimum temperatures for each day. We also calculated the number of days below freezing that each tree experienced during flowering (March 23–April 27), during seed development (April 28–May 17), as well as the mean daily temperature and growing degree days during flowering and seed development. These weather variables were included as explanatory variables in the models described below. All data analyses were done in R v4.0.0 (RCoreTeam, 2020).

The five dependent variables that we wanted to predict were (a) the probability of producing seeds, (b) the total number of seeds, (c) the proportion of seeds with an embryo present, (d) mean embryo size, and (e) germination success. The explanatory variables that we tested for all models included hemlock density, stand density, individual tree age, height, and canopy volume, as well as various temperature and growth indicators. There were three potential growth indicators (BAI3, BAI5, and DBH), and four potential temperature indicators (growing degree days and number of frost days, during flowering and seed development). Because the growth indicators (and the temperature indicators) were all highly correlated, only one could be included at a time. We did not include elevation as an explanatory variable, since elevation was used to extrapolate the temperature variables from the data loggers, to the individual tree. Model selection was done with the library MuMIn (Barton, 2020) and was based on the lowest AICc score, weights and the amount of deviance/variance explained. All results presented below are for the final chosen model.

For each tree, the number of depredated seed and fully formed seed was combined into “total seed” as depredated seed were also fully formed and would have had the potential to contribute to the number of viable seed if it had not been eaten. To determine which variables influenced the probability of a tree producing seed, a logistic regression was used with seed production (0 or 1) as the response variable. Frost days during flowering and GDD during flowering were both tested as temperature indicators but neither were included in the final model. A growth indicator was also not included in the final model. The final model included canopy volume and age.

Total seed production was modeled with a Poisson distribution. Over dispersion was corrected using a quasi‐GLM model. Frost days during flowering and GDD during flowering were both tested as temperature indicators. The model that explained the highest amount of deviance was selected as the final model and included stand density, frost‐free days during flowering and BAI3 as explanatory variables.

The proportion of seeds filled per individual tree was modeled using a quasibinomial distribution. The proportion of seed filled was calculated for each tree by dividing the number of seeds with embryo presence by the total number of seeds that were scanned. These models were also weighted by the number of seeds that were scanned per tree (i.e., not all trees had 25 seeds scanned). Frost‐free days during flower, GDD during flower, frost‐free days during seed, and GDD during seed were all tested as temperature indicators. The final model did not include any temperature indicators, and only included BAI5 and height as explanatory variables.

The linear model for predicting average embryo size per tree (mm^3^) was also weighted by the number of seeds that had embryo presence per tree. Frost‐free days during seed and GDD during seed were tested as temperature indicators but were not included in the final model. The final model included DBH as a growth indicator, as well as height and hemlock density.

Using the R package vegan (Oksanen et al., 2019), a principle component analysis (PCA) was run at the individual seed level to visualize the relationship between seed morphology and germination. A permutational multivariate analysis of variance (PERMANOVA) was used to test if the groups (germinated vs. did not germinate) were associated with significantly different seed morphology. A second PCA was performed at the tree level using the mean seed morphology measurements for each tree. From this second PCA, PCA axis one was extracted and used as an explanatory variable in a quasibinomial model, where the response variable was the proportion of seeds that germinated out of the seeds with embryos present. This model was weighted by the number of seeds with embryo presence. We also tested embryo volume in addition to the other explanatory variables (as above). The final model included embryo volume alone, as the sole predictor for germination success.

## 3 RESULTS

The mean age of all the study trees was 96 years (±1.6 *SE*), and the average DBH was 27 cm (±1.4 *SE*). DBH and age were not correlated (Spearman's Rank *r* = .08, *n* = 86, *p* = 0.45). The total number of seeds collected per tree ranged from 0 to 950, with a mean of 139 (±33.02 *SE*). On average, 20% (±3% *SE*) of the seeds collected per seed‐bearing tree were aborted, and 27% (±4% *SE*) of the collected seeds per tree showed signs of predation. Aborted seeds were usually collected as a pair, still attached to their partner. The total number of seeds collected was positively correlated with the number of aborted seeds (Spearman's Rank *r* = 0.77, *n* = 35, *p* < 0.001) and also with the number of predated seeds (Spearman's Rank *r* = 0.86, *n* = 35, *p* < 0.001).

### 3.1 Probability of producing seed

**FIGURE 2:**
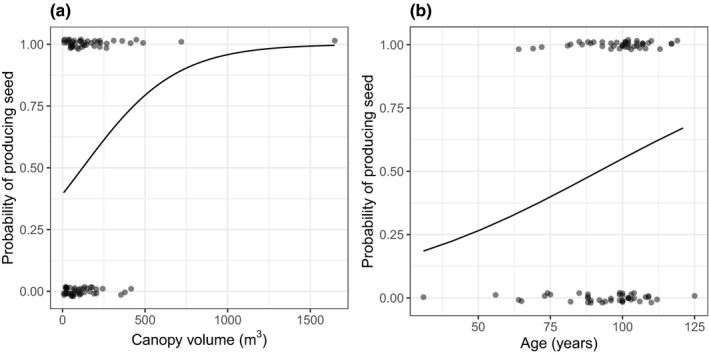
The probability that a tree will produce seed as a function of (a) canopy volume and (b) age. Each point represents a single tree, and the line shows the prediction from a multiple logistic regression. The points have been jittered on the *y*‐axis only, to better visualize the individual points

Out of the 44 trees that produced seed, 13 were scored as a 3 (i.e., >80% of canopy having seed), 15 were scored as a 2 (i.e., 20%–80% of the canopy having seed), and 16 were scored as a 1 (i.e., <20% of the canopy having seed). Seed traps were successful at catching seeds for 35 of the 44 seed‐bearing trees. The nine remaining trees that we were unable to collect seeds from were all trees that had been scored as a 1. The only variable that was significant for predicting the probability that a tree produced seed (i.e., seed score >0) was canopy volume (Table 1; Figure 2a). Trees with larger canopies were more likely to produce seeds. One very large individual appeared to have a strong influence on the results; however, the relationship between seed production and canopy volume remains even when this one individual is removed (see Table S1, Figure S1). Age was retained in the final model even though it was not statistically significant (Table 1), with older trees being more likely to produce seeds (Figure 2b).

**TABLE 1:** Results of a multiple logistic regression, predicting the probability of producing seed as a function of canopy volume and age. Predictor variables were standardized. Thus, the odds ratio represent the increase in likelihood of producing seed (i.e., OR > 1.0) per one standard deviation increase in canopy volume, or age. See Figure 2 for data and fitted model results

	Odds ratio	*df*	Deviance	*p*
Canopy volume	2.08	1	4.42	0.04
Age	1.44	1	2.47	0.12
Residuals		85	111.61	

### 3.2 Total seed production

**FIGURE 3:**
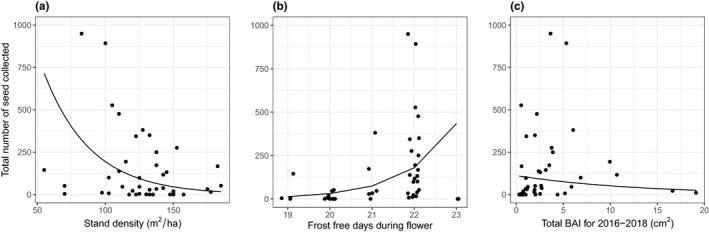
The total number of seeds collected per tree, as a function of (a) stand density, (b) number of frost‐free days during flower, and (c) basal area increment (BAI) sum for the past three years. Each point represents a single tree, and the line shows the prediction from a multiple generalized linear regression with a Poisson distribution. For (b), the points have been jittered on the *x*‐axis only, to better visualize the individual points

The final model for predicting the total number of seeds collected included stand density, the number of frost‐free days during flowering, and a growth indicator (BAI3; Figure 3). The strongest predictor was the number of frost‐free days during flowering (Table 2). Trees that experienced fewer frost days during flowering had significantly greater total seed production (Figure 3b). This relationship was strong even with the relatively small difference in the number of frost‐free days (19–23 days) between individuals. For the trees that experienced the fewest frost‐free days (19), we collected a mean of 38 seeds (±35.6 *SE*). For the trees that experienced the most frost‐free days (22 days), we collected a mean of 236 seeds (±57 *SE*). Stand density was also a strongest predictor of the total number of seeds collected per tree (Table 2), as trees in lower density stands were significantly higher seed producers (Figure 3a). Finally, there was a negative relationship between the total number of seeds collected and growth (i.e., sum of the BAI for the past 3 years) (Figure 3c). The final model explained 41% of the deviance in the total number of seeds collected.

**TABLE 2:** Results from a generalized linear regression, using a quasi‐Poisson distribution to predict the total number of seeds. The final model included stand density, frost‐free days during flower (i.e., temperature >0°C), and total basal area increment (BAI) for the past 3 years. Predictor variables were standardized. See Figure 3 for data and fitted model results

	Scaled estimate	Scaled *SE*	*df*	Deviance	*F*	*p*
Stand density	−0.82	0.20	1	2,940	19.3	<0.001
Frost‐free days during flowering	1.00	0.23	1	3,759	24.7	<0.001
BAI_3_	−0.31	0.19	1	536	3.5	0.07
Residuals			43	6,082		

### 3.3 Proportion of seeds with embryo development

**FIGURE 4:**
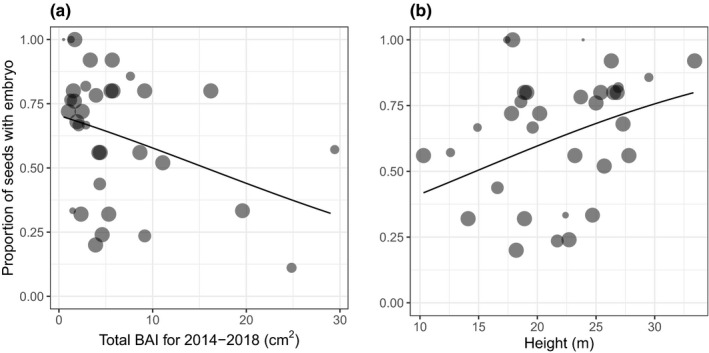
The proportion of scanned seeds that had an embryo present, per tree, as a function of (a) basal area increment (BAI) sum for the past five years and (b) height. Each point represents a single tree, with the size of the point corresponding to the number of seeds scanned per tree (i.e., the largest points had the maximum 25 seeds scanned per tree; see Figure 4 for the legend). The line shows the prediction from a multiple generalized linear regression with a binomial distribution, weighted by the number of seeds scanned per tree

Out of the 661 scanned seed, only 63% (415 seeds) showed embryo development. Within a tree, the mean proportion of seeds that had an embryo was 61% (±4.5% *SE*); however, this ranged from 0% to 100% depending on the individual. The only variable that was significant for predicting the proportion of seeds with embryos per tree was height (Table 3). Taller trees had a significantly higher proportion of seeds with an embryo (Figure 4b). Although total BAI for the last 5 years of growth was not statistically significant, the final model included it as an explanatory variable (Table 3, Figure 4a). The trend was for trees with a lower total BAI for the last 5 years to have a higher proportion of seeds with embryos.

**TABLE 3:** Results from a generalized linear regression, using a quasibinomial distribution to predict the proportion of seeds per tree with embryo present. The final model included total basal area increment (BAI) for the past 5 years and height. Predictor variables were standardized. See Figure 4 for data and fitted model results

	Scaled estimate	Scaled *SE*	*df*	Deviance	*F*	*p*
BAI_5_	−0.38	0.24	1	11.0	2.5	0.13
Height	0.39	0.18	1	19.3	4.3	0.05
Residuals			33	134.2		

### 3.4 Embryo volume

**FIGURE 5:**
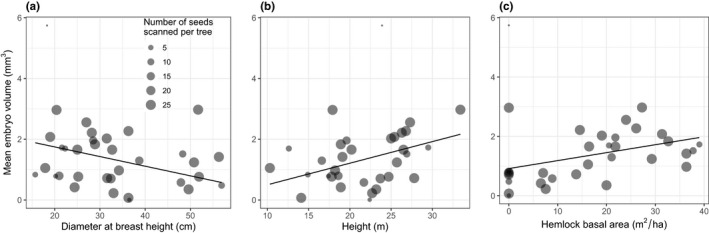
Mean embryo volume per tree, as a function of (a) diameter at breast height (DBH), (b) height, and (c) hemlock basal area surrounding the tree. Each point represents a single tree, with the size of the point corresponding to the number of seeds scanned per tree (i.e., the largest points had the maximum 25 seeds scanned per tree). The line shows the prediction from a multiple linear regression, weighted by the number of seeds per tree

Mean embryo volume was 1.37 mm^3^ (±0.075 *SE*). Per tree, mean embryo volume was influenced by DBH, height, and hemlock basal area in the surrounding stands (Table 4). Mean embryo volume per individual was larger in trees with a smaller DBH (Figure 5a), taller (Figure 5b) and those that had more hemlock surrounding the tree (Figure 5c).

**TABLE 4:** Results from a multiple linear regression, to predict the mean embryo volume of seeds per tree (*n* = 35 trees). Predictor variables were standardized. See Figure 5 for data and fitted model results

	Scaled estimate	Scaled *SE*	*t*	*p*	*R* ^2^
DBH	−0.39	0.15	−2.7	0.007	0.32
Height	0.37	0.14	2.7	0.007	
Hemlock density	0.36	0.14	2.5	0.01	

### 3.5 Germination success and seed morphology

**FIGURE 6:**
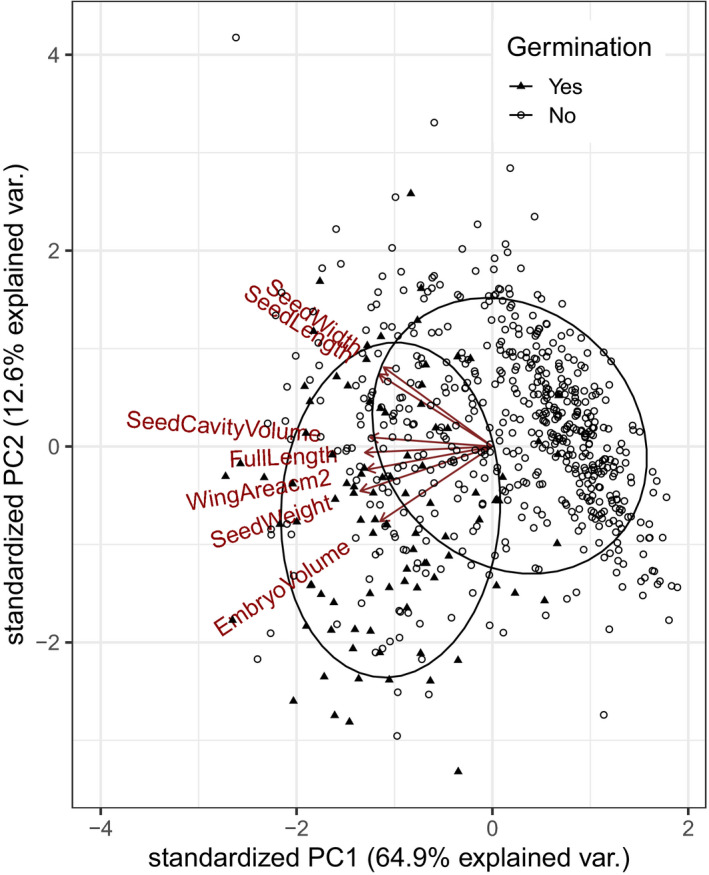
Principle component analysis (PCA) using seed morphology measurements. Each point represents a single seed with larger seeds separating out to the left. Ellipses show the 95% confidence intervals for each group. Seeds that germinated versus those that did not germinate are significantly different (PERMANOVA, *F*_1,656_ = 102.7, *p* = 0.001)

**FIGURE 7:**
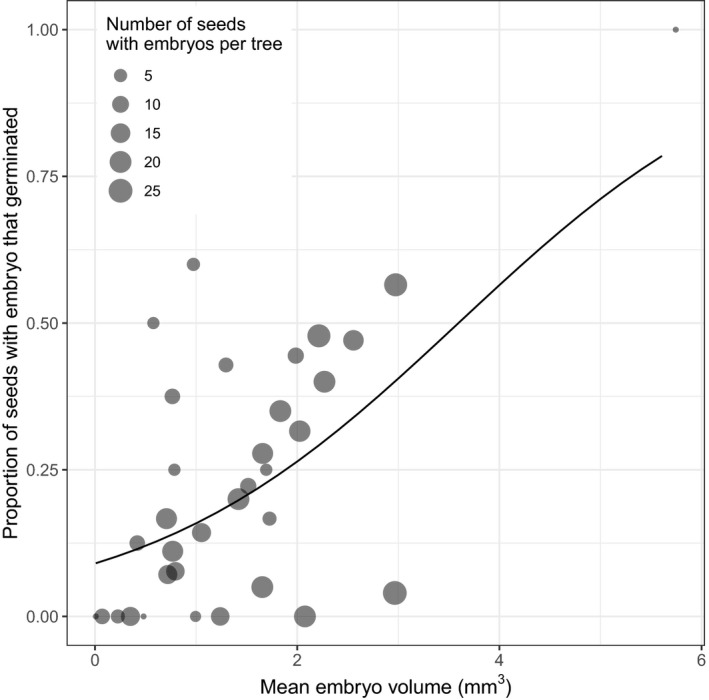
The proportion of seeds with embryo present that successfully germinated as a function of mean embryo volume, per tree. Each point represents a single tree, with the size of the point corresponding to the number of seeds per tree. The line shows the prediction from a generalized linear regression with a quasibinomial distribution, weighted by the number of seeds with embryos per tree

Only 95 seeds successfully germinated. This represents a germination success of 23% of the seeds that had an embryo present (i.e., based on the seed scan). This low germination rate was partly due to issues with mold, and potentially because our cold stratification treatment was not long enough to completely break dormancy. Using a PCA to visualize the seed morphology measurements, we found that PCA axis 1 was correlated to size and explained 64.9% of the variance (Figure 6). PCA axis 2 only explained 12.6% of the variance and was not strongly correlated to any one particular morphological characteristic. Seeds that were larger in all morphological characteristics (i.e., embryo volume, weight, wing area, seed length, seed width, and length of the full samara) were the most likely to germinate, and there was a significant difference in morphology between those seeds that did germinate, and those seeds that did not (PERMANOVA, *F*_1,656_ = 102.7, *p* = 0.001; Figure 6). We found the same pattern when we ran a PCA using the mean morphology characteristics per tree (Figure S2), with PCA axis 1 being correlated with size and explaining almost all of the variance (85% of the variance). To test what variables influenced the proportion of seeds with embryos that successfully germinated per tree, we used PCA axis 1 representing the mean tree seed morphology (Figure S2), as well as temperature variables, individual characteristics, and stand information to explain the proportion of seeds that had embryo presence that germinated. PCA axis 1 was not significant (*F*_1,33_ = 2.74, *p* = 0.11, deviance explained = 6.4, residual deviance = 95.2) for predicting germination. The only variable which was significant to explain the proportion of seeds with embryos that germinated was mean embryo volume (*F*_1,33_ = 9.95, *p* = 0.003, deviance explained = 20.1, residual deviance = 95.2; Figure 7). With the exception of mean seed weight (which was highly correlated with mean embryo volume; Pearson's *r* = 0.95, *n* = 34, *p* < 0.001), none of the other seed morphology measurements were significant (Table S2).

## 4 DISCUSSION

**FIGURE 8:**
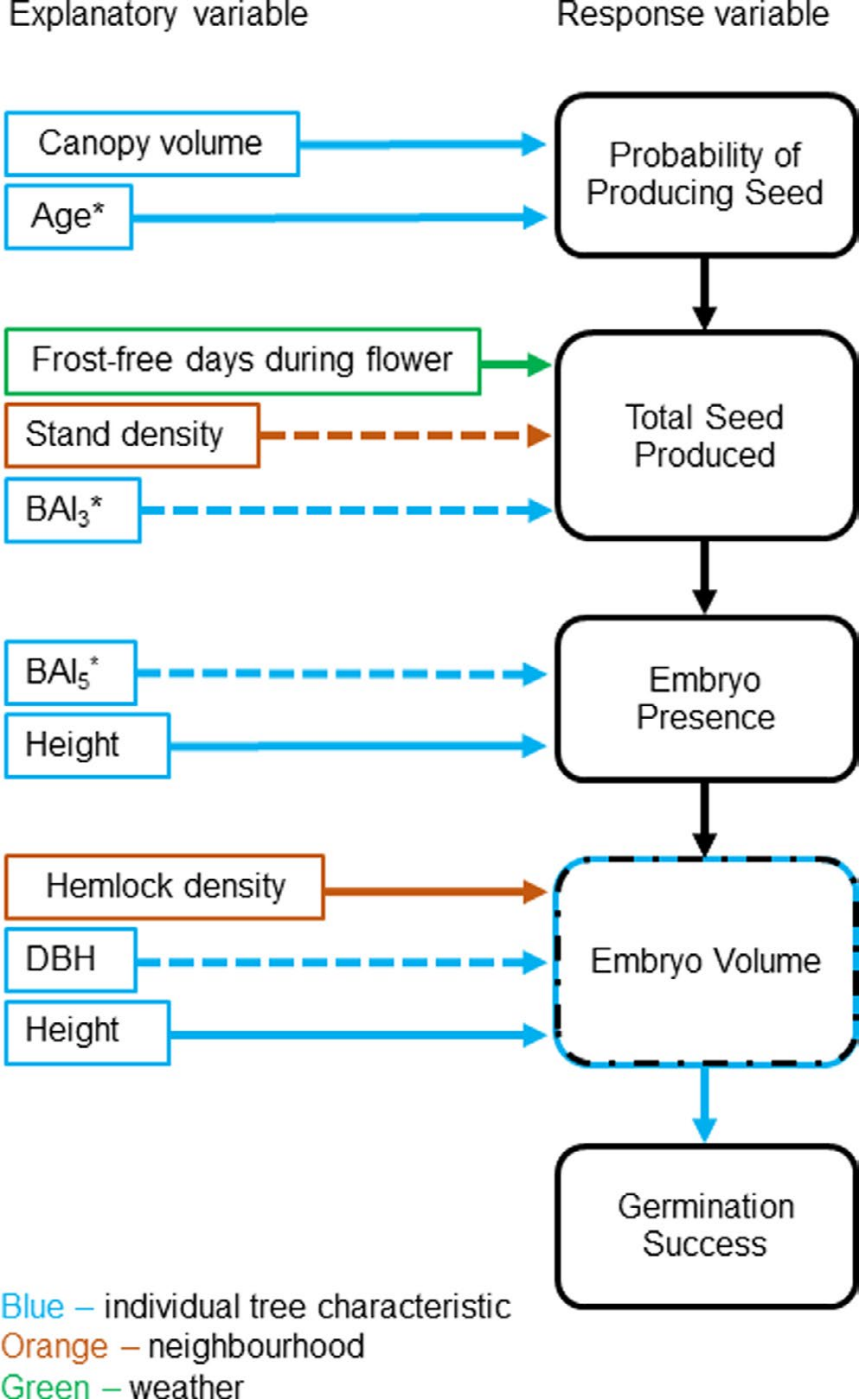
Summary diagram illustrating how each stage of reproduction is influenced by different explanatory variables. Solid lines indicate the explanatory variable had a positive effect; dotted lines indicate a negative effect. Starred variables were not statistically significant but were included in the final model and are biologically relevant. Embryo volume has a dashed blue/black box to indicate its role as both a response variable and an individual tree characteristic

Understanding how individual and environmental factors affect reproductive success is necessary for predicting how plants will respond to a changing climate. A more complete understanding will come from examining all stages of reproduction, including what makes a tree likely to produce seed, what impacts the total number of seed, and what impacts seed quality (i.e., embryo size and germination success). Based on the results of the current study, weather directly affects the total number of seeds produced but does not have a direct effect on seed quality (Figure 6). Instead, we found it was mostly individual traits and some stand characteristics that determined differences in seed quantity and quantity per tree (Figure 8). Below we will discuss in more detail how reproduction in red maple is influenced by (a) weather, (b) individual characteristics, and (c) resources.

### 4.1 Weather and reproduction

For red maple, the only relationship between weather and reproduction that could be identified was a reduction of seed number in response to frost events during flowering. This is consistent with a study done by Tremblay et al. (2002) where the frequency of late spring frosts also had a negative impact on seed production in red maple. The reduction in seed number may be caused by one or a combination of the following processes: (a) frost damaged flowers may be incapable of being fertilized, (b) low temperatures can negatively affect pollen viability and pollen tube growth, and (c) pollinator activity may be reduced in low temperatures.

The importance of temperature for successful fertilization in plants has been previously recorded (e.g., Luza et al., 1987; Owens, 1992; Pigott & Huntley, 1981). Cold temperatures stunt and slow down pollen tube growth, which reduces the likelihood of fertilization as the style, slowly degrades over time (Owens, 1992). Some species, such as *Tilia cordata*, *Juglans regia*, and *Juglans nigra*, have a critical temperature threshold, where pollen tube growth completely fails if temperatures drop below 15°C (Luza et al., 1987; Pigott & Huntley, 1981). In addition to the actual process of fertilization, cold temperatures also affect pollinator activity. While wind is the main mechanism for pollination in red maple, insects, such as bees and flies, are known to visit the flowers (Batra, 1985). Although the effectiveness and frequency of insect pollination in red maple has not been documented, insect pollinators are less likely to fly in cold temperatures or bad weather conditions (Corbet et al., 1993).

In both of the study sites, the terrain was topographically diverse. This created an environment where trees that were geographically close could experience a different number of frost days. Even a minor difference in frost days between trees had a large impact on the total number of seeds collected (Figure 3b). Frost events are also known to stimulate parthenocarpy in fruit trees (i.e., fruit development without ovule fertilization; Goldwin, 1992; Lewis, 1942). However, in this study, no measure of temperature (i.e., frost days during flower, frost days during seed, or growing degree days) was predictive for embryo presence.

While we did not observe a direct effect of climate on seed quality, other studies have demonstrated that temperature and/or moisture do directly affect the viability of seed for some species. For example, *Tilia cordata's* seed viability is strongly influenced by temperature while *Acer pseudoplatanus* seed viability is sensitive to moisture (Pigott & Huntley, 1981; Pigott & Warr, 1989). Another study of black spruce (*Picea mariana*) found no correlation between cone number and elevation but did see a reduction in seed quality at higher elevations in response to lower heat sums (Sirois, 2000). In addition to the direct effect of climate on reproduction, climate may indirectly influence seed quantity and quality by influencing stand characteristics and individual traits. Both of which are important for reproduction in red maple (see section below). For example, increased temperature and moisture increased basal area increment in red maple (Zhang et al., 2015). When conditions favor growth, then reproduction may be delayed, however; this delayed reproduction may lead to increased reproduction later in life (Wolgast & Zeide, 1983). Climate change will lead to longer growing seasons and warmer temperatures at species range limits, which may allow trees to accumulate more resources that could be allocated to reproduction (Zhang et al., 2015).

Climate also changes stand dynamics. As the climate changes we can expect to see more severe weather events (e.g., hurricanes and droughts) as well as more mortality caused by biotic agents (e.g., bark beetles) (Bolte et al., 2009; Ruiz‐Benito et al., 2013). All of which will cause increased mortality, changes in stand density, and competition. It has been suggested that red maple is likely to benefit, indirectly, from a changing climate because of these changes to disturbance regimes (Zhang et al., 2015). Red maple responds positively to disturbances that create large canopy gaps (i.e., increase light availability) and thin stands, creating a dense regeneration layer. Indeed, red maple did produce more seed in areas of low stand density in our study. These trees were likely benefiting from reduced competition (i.e., more resources were available for reproduction). Thus, it is likely that red maple reproduction and recruitment will benefit as large disturbances become more common in the future.

### 4.2 Individual characteristics and reproduction

We found individual tree characteristics to be important at every stage of tree reproduction (Figure 8). Individuals with large canopy volumes were more likely to produce seeds. As canopy volume relates to potential photosynthetic capacity, this result is not unexpected. These individuals should have more carbohydrate resources to direct toward reproduction (Jackson & Palmer, 1977). We also found older trees more likely to produce seeds, which is also consistent with the literature (Primack et al., 1986).

Reproductive effort (or total number of seed) is also thought to be positively correlated with age and/or size (Viglas et al., 2013). However, we did not find a significant positive relationship between seed production and age or DBH. Surprisingly, we found a negative relationship between DBH and seed quality (Figure 5a). Trees with smaller DBH and trees that were taller produced larger embryos, and larger embryos are more likely to germinate (Figure 5). Overall, this meant that smaller trees (at least in terms of diameter) had the highest seed quality. The absence of an age effect on total seed production may be due to the relatively even age distribution in our stands. Almost half of the trees (46%) were between 100 and 110 years old, and the difference between the oldest and the youngest tree was only 69 years.

In our study, it was height as well as total growth during the last 3–5 years that was a strong predictor of both seed quantity and quality. Taller trees had a higher proportion of their seeds filled and had larger embryos. Red maple samaras are one of the many seeds that are able to produce their own photosynthate (i.e., the samara wings are photosynthetically active). Taller trees may have more light reaching the ripening fruit enabling them to produce seeds with larger embryos at less cost to the tree (Bazzaz, 1979; Greene & Johnson, 1992). The reduced growth of trees who produced higher quantity and quality of seeds (Figure 3c, Figure 4a) is consistent with many dendroecological studies that show reduced growth rates in years of high reproduction (e.g., Koenig & Knops, 1998; Obeso, 1997). This is likely because producing fruit is more expensive than flowers and being female is associated with additional reproductive costs (Primack et al., 1986) resulting in decreased growth.

While not quantified in this study, the role of genetics in reproduction cannot be overlooked. Genetic differences can affect both seed number and seed morphology (Donohue et al., 2005), and common garden experiments have shown that populations demonstrate morphological adaptations related to their local climate (reviewed in Aitken et al., 2008). Thus, differences in seed number and quality between plants may also reflect genetics and individual adaptations to local selection pressures.

### 4.3 Resources and reproduction

In general, increased availability of soil nutrients is associated with higher reproductive capacity and nitrogen, specifically, is important for embryo growth (reviewed in Heslop‐Harrison, 1957; Lyndon, 1992). Although we did not measure any soil variables, we found an unexpected positive correlation between embryo volume and hemlock basal area in the surrounding stand (Figure 5c). Several potential processes could explain this pattern. First, the shift from mixed hardwood forest into a hemlock dominated area usually implies that there are differences in soil conditions. Hemlocks are associated with ectomycorrhizal fungi (ECM), while red maple is associated with arbuscular mycorrhizal fungi (AM). Typically, areas that are dominated by ECM‐associated species have slow nutrient regeneration and low mineralization rates (i.e., less nitrogen is available for plants) when compared to areas where AM species dominate (Binkley & Giardina, 1998). However, the dominance of hemlock would imply reduced nutrient availability, and thus, we might expect embryo size in red maple to decrease in these areas. However, this is exactly opposite to what was observed. Thus, any direct effect of hemlock's impact on nutrient availability on red maple reproduction is not a likely explanation. One possible explanation is reduced competition for nutrients between hemlock and red maple, as they have different ways of acquiring nutrients (i.e., different mycorrhizal associations) (Chen et al., 2016). In areas containing a higher density of other AM‐associated species, red maple may be in direct competition with those species for resources.

Alternatively, the perceived association between hemlock density and red maple embryo size may not be related to hemlock at all. Red maple and sugar maple (*Acer saccharum*) have a bimodal distribution, with sugar maple occurring more frequently in nutrient‐rich sites and red maple occupying nutrient‐poor sites (Abrams, 1998). Studies of sugar maple in hemlock stands have shown that the nitrogen limitation suppresses their regeneration (Binkley & Giardina, 1998). Our final suggestion is that red maple was being outcompeted by sugar maple in those areas that did not have hemlocks, resulting in smaller embryos. The exclusion of sugar maple in the areas with high hemlock density allowed red maple to put more energy into reproduction.

## 5 CONCLUSION

This study provides evidence that the presence of reproductively mature trees is not a good indicator of a population's reproductive capacity. Furthermore, we demonstrate that looking at total seed production alone is not sufficient, as a tree can produce many seeds but only a small portion of them may contain viable embryos. To understand why we are seeing tree range contractions instead of expansions (e.g., Zhu et al., 2012), understanding the factors controlling reproductive effort, seed quality, and germination are critical. It is uncertain how ongoing and future climate change will ultimately affect red maple reproduction. While warmer springs should cause fewer frost days for these populations located at the edge of their current ranges, increasing climate variability has the potential to lead to more spring frosts, even though the average temperature increases. This could negatively affect total seed availability and reduce range expansions at the northern edge. However, seed quality and germination success were mostly determined by individual and stand‐level characteristics, such as stand composition, height, growth, and size of individual trees. Thus, a combination of climate, stand‐ and individual‐level characteristics should be included when predicting the likelihood of seed reaching new areas and successfully establishing.

## CONFLICT OF INTEREST

The authors have no competing interests to declare.

## AUTHOR CONTRIBUTION

**Abigail R Goszka:** Conceptualization (equal); Formal analysis (equal); Methodology (lead); Writing‐original draft (equal); Writing‐review & editing (equal). **Rebecca S. Snell:** Conceptualization (equal); Formal analysis (equal); Methodology (supporting); Writing‐original draft (equal); Writing‐review & editing (equal).

## Supporting information

Appendix S1

## Data Availability

All data and associated R scripts are available on Dryad https://doi.org/10.5061/dryad.2rbnzs7m3
