# Supplementary material for: Seed quality and seed quantity in red maple depends on weather and individual tree characteristics
Source: Ecol Evol. 2020 Oct 14;10(23):13109–21. doi: 10.1002/ece3.6900 (PMC7713923; doi:10.1002/ece3.6900)
Supplement: Supplementary file 1 — Appendix S1 [file ECE3-10-13109-s001.docx]

**Appendix**

**Seed quality and seed quantity in red maple depends on weather and individual tree characteristics,** A. R. Goszka and R. S. Snell

Results from the probability of producing seed, if we remove the tree with the largest canopy (see Table 1, Figure 2 in the manuscript for comparison).

**Table S1.** Results of a multiple logistic regression, predicting the probability of producing seed as a function of canopy volume and age. Predictor variables were standardized, to remove the units. Thus, the odds ratio represent the increase in likelihood of producing seed (i.e., OR > 1.0) per one standard deviation increase in canopy volume, or age. The tree with the largest canopy volume has been removed.

|  | Odds ratio | df | Deviance | P |
| --- | --- | --- | --- | --- |
| Canopy volume | 1.57 | 1 | 3.75 | 0.05 |
| Age | 1.43 | 1 | 2.46 | 0.12 |
| Residuals |  | 84 | 111.61 |  |


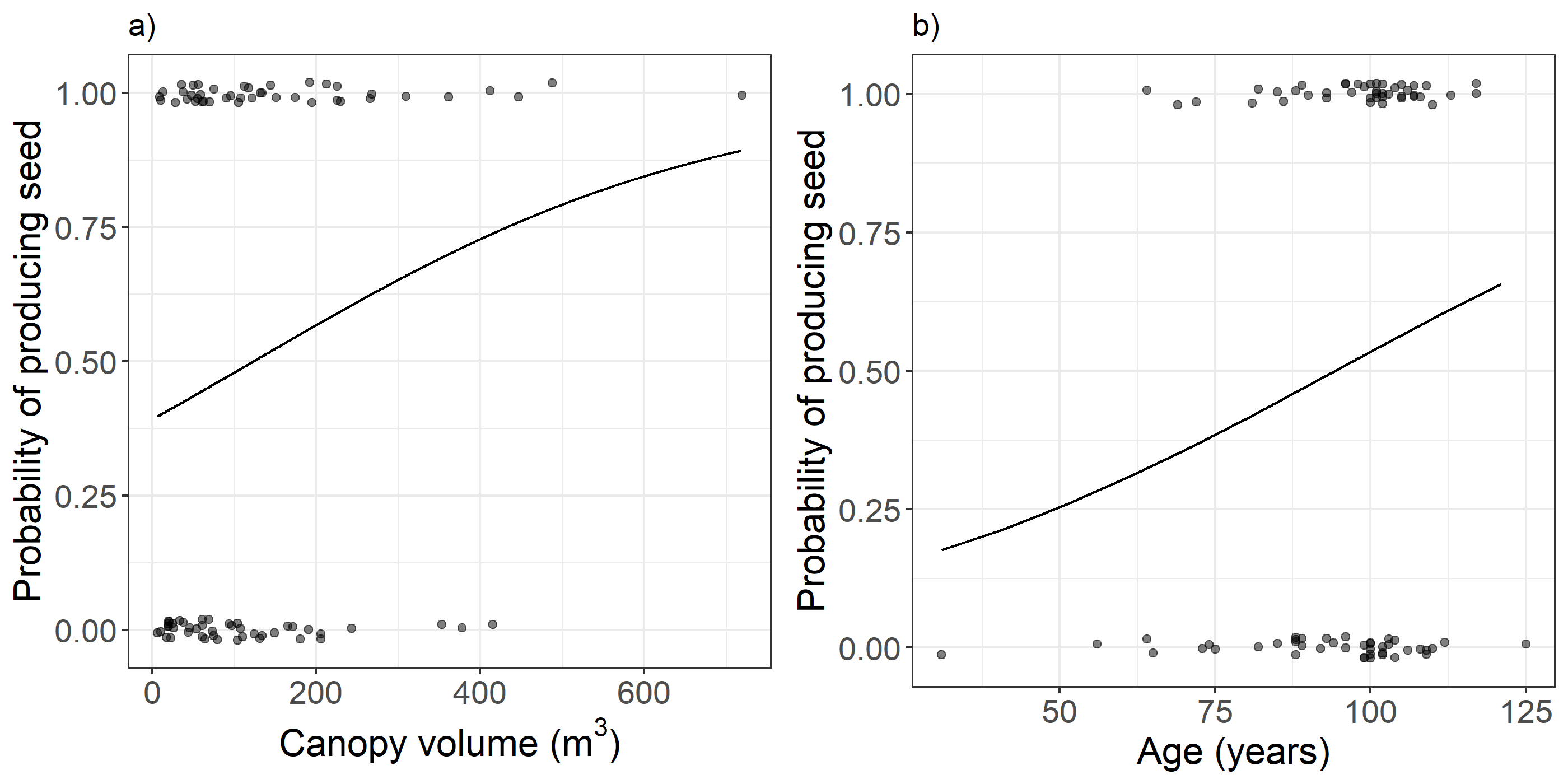


**Figure S1.** The probability that a tree will produce seed as a function of (a) canopy volume and (b) age. Each point represents a single tree and the line shows the prediction from a multiple logistic regression. The points have been jittered on the y axis only, to better visualize the individual points.


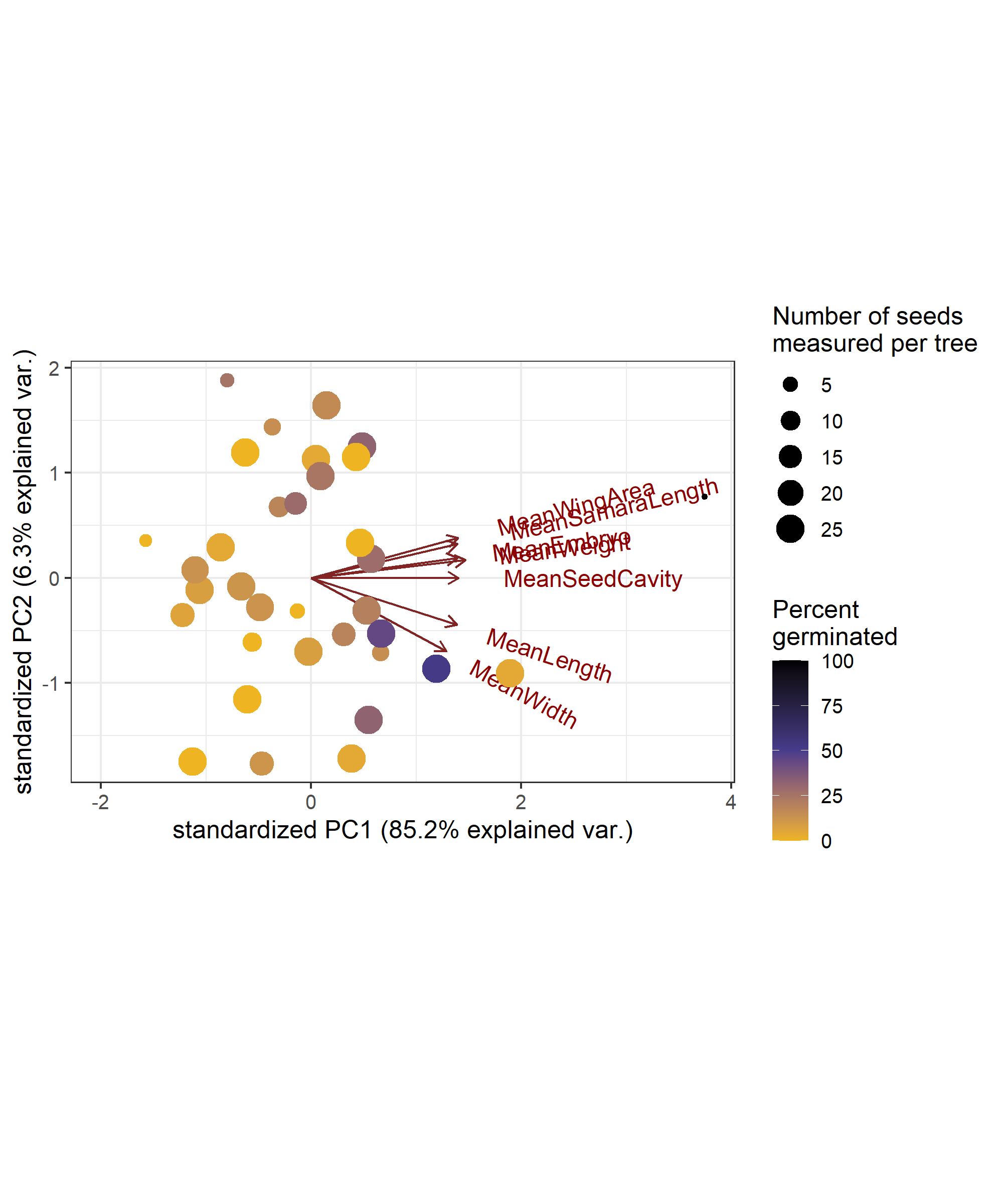


**Figure S2.** Principle Component Analysis (PCA) using the mean seed morphology measurements per tree. Each point represents a single tree, with the size of the point corresponding to the number of seeds scanned per tree (i.e., the largest points had the maximum 25 seeds scanned per tree). The colour represents how many of the scanned seeds, successfully germinated. PCA axis 1 was extracted, and used as a predictor in a generalized linear model, to predict germination success (see Results in main manuscript).

**Table S2.** Results from quasibinomial regressions, predicting the proportion of seeds with embryo present that successfully germinated per tree, as a function of different seed morphology measurements. PCA axis 1 included all measures of seed morphology (Figure S2) was not significant, so each measure of seed morphology was tested one at a time, to see if a singular seed morphology measurement was significant for predicting germination. Results for PCA axis 1 and mean embryo volume are reported in the main manuscript. For all model, null deviance = 95.2 on 33 df.

|  | Deviance explained | F | P |
| --- | --- | --- | --- |
| PCA axis 1 | 6.39 | 2.74 | 0.11 |
|  |  |  |  |
| Mean embryo volume | 20.07 | 9.95 | 0.003 |
|  |  |  |  |
| Mean seed weight | 11.47 | 5.17 | 0.03 |
|  |  |  |  |
| Mean seed length | 3.70 | 1.53 | 0.22 |
|  |  |  |  |
| Mean wing area | 2.92 | 1.19 | 0.28 |
|  |  |  |  |
| Mean samara length | 0.59 | 0.23 | 0.63 |
|  |  |  |  |
| Mean seed cavity | 3.42 | 1.39 | 0.25 |
